# Mycorrhizal Symbiotic Efficiency on C_3_ and C_4_ Plants under Salinity Stress – A Meta-Analysis

**DOI:** 10.3389/fmicb.2016.01246

**Published:** 2016-08-11

**Authors:** Murugesan Chandrasekaran, Kiyoon Kim, Ramasamy Krishnamoorthy, Denver Walitang, Subbiah Sundaram, Manoharan M. Joe, Gopal Selvakumar, Shuijin Hu, Sang-Hyon Oh, Tongmin Sa

**Affiliations:** ^1^Department of Environmental and Biological Chemistry, Chungbuk National UniversityCheongju, South Korea; ^2^Department of Microbiology, School of Life Sciences, Vels UniversityChennai, India; ^3^Department of Plant Pathology, North Carolina State University, RaleighNC, USA; ^4^Department of Animal Sciences, North Carolina Agricultural and Technical State University, GreensboroNC, USA

**Keywords:** arbuscular mycorrhizal fungi, salinity stress, meta-analysis, C_3_ and C_4_ plants, nutrient uptake, plant biomass

## Abstract

A wide range of C_3_ and C_4_ plant species could acclimatize and grow under the impact of salinity stress. Symbiotic relationship between plant roots and arbuscular mycorrhizal fungi (AMF) are widespread and are well known to ameliorate the influence of salinity stress on agro-ecosystem. In the present study, we sought to understand the phenomenon of variability on AMF symbiotic relationship on saline stress amelioration in C_3_ and C_4_ plants. Thus, the objective was to compare varied mycorrhizal symbiotic relationship between C_3_ and C_4_ plants in saline conditions. To accomplish the above mentioned objective, we conducted a random effects models meta-analysis across 60 published studies. An effect size was calculated as the difference in mycorrhizal responses between the AMF inoculated plants and its corresponding control under saline conditions. Responses were compared between (i) identity of AMF species and AMF inoculation, (ii) identity of host plants (C_3_ vs. C_4_) and plant functional groups, (iii) soil texture and level of salinity and (iv) experimental condition (greenhouse vs. field). Results indicate that both C_3_ and C_4_ plants under saline condition responded positively to AMF inoculation, thereby overcoming the predicted effects of symbiotic efficiency. Although C_3_ and C_4_ plants showed positive effects under low (EC < 4 ds/m) and high (>8 ds/m) saline conditions, C_3_ plants showed significant effects for mycorrhizal inoculation over C_4_ plants. Among the plant types, C_4_ annual and perennial plants, C_4_ herbs and C_4_ dicot had a significant effect over other counterparts. Between single and mixed AMF inoculants, single inoculants *Rhizophagus irregularis* had a positive effect on C_3_ plants whereas *Funneliformis mosseae* had a positive effect on C_4_ plants than other species. In all of the observed studies, mycorrhizal inoculation showed positive effects on shoot, root and total biomass, and in nitrogen, phosphorous and potassium (K) uptake. However, it showed negative effects in sodium (Na) uptake in both C_3_ and C_4_ plants. This influence, owing to mycorrhizal inoculation, was significantly higher in K uptake in C_4_ plants. For our analysis, we concluded that AMF-inoculated C_4_ plants showed more competitive K^+^ ions uptake than C_3_ plants. Therefore, maintenance of high cytosolic K^+^/Na^+^ ratio is a key feature of plant salt tolerance. Studies on the detailed mechanism for the selective transport of K in C_3_ and C_4_ mycorrhizal plants under salt stress is lacking, and this needs to be explored.

## Introduction

Approximately 70% of the farm soils on earth are either salt-affected or subjected to salinity (Munns and Tester, 2008). Furthermore, it has been predicted that 30% of the cultivable soils will become unusable by 2050 due to salinity, and this warrants appropriate salt alleviating technologies for sustaining food production (Wang et al., 2003). Plants can be divided in to three different types in terms of the way in which they use photosynthesis, C_3_, C_4_, and CAM. The difference between these types is how plant uses carbon dioxide in the growth process. Although C_4_ plants are relatively few, i.e., 3% of plant species compared with the much more numerous C_3_ plants (∼7500 C_4_ species to nearly ∼250000 C_3_ species), they account for approximately 30% of primary productivity (Sage and Zhu, 2011). As described previously in detail, Zhu et al. (2008) and de Bossoreille de Ribou et al. (2013) reported that the conversion efficiency (from solar energy to biomass) during photosynthesis is 4.6% for C_3_ and 6% for C_4_ plants. Thus, global productivity has to better exploit the superior engine of C_4_ photosynthesis, both by using it on a greater scale (i.e., engineer the C_4_ photosynthetic pathway into C_3_ plants) and by improving the ability of C_4_ plants to resist environmental stress (Hibberd et al., 2008; Kajala et al., 2011; de Bossoreille de Ribou et al., 2013). Among the major food crops, C_3_ and C_4_ plants are widely studied for their response to salt stress (Stepien and Klobus, 2005; Chaves et al., 2009; Ivlev et al., 2013). Mane et al. (2011) reported a higher shoot/root ratio at 300 mM NaCl in the C_4_ plant Bajra, in which plant biomass production was increased by 29.17%. In C_4_ maize plants, shoot and root growth was not impacted up to 1.5 g NaCl/Kg dry substrate (Sheng et al., 2008). Conversely, shoot and root growth of C_3_ plants wheat (Mohammad et al., 1998) and tomato (Datta et al., 2009) were decreased proportionately with the increase in NaCl concentration up to 125 mM. Earlier researchers (Omoto et al., 2012; Ivlev et al., 2013) have attributed this successful salt-tolerance effect in C_4_ plants to the CO_2_ concentrating mechanism of these plants under stress conditions.

Symbiotically associating microorganisms have been widely used for modulating stress factors by increasing the nutrient availability and sustaining productivity in these plants (Dodd and Perez-Alfocea, 2012; Pellegrino et al., 2015). Symbiotically associated and soil inhabiting arbuscular mycorrhizal fungi (AMF) have been widely studied for their effective scavenging of soil nutrients, owing to their larger surface root volume (Parniske, 2008). The symbiosis between plant roots and AM fungi is one of the best plant’s strategies for growing under salt stress (Evelin et al., 2009; Porcel et al., 2012). AM fungi have been frequently reported to improve host plants’ tolerance to salt stress. Improved salt tolerance following mycorrhizal colonization may be the result of a more efficient nutrient uptake (Porras-Soriano et al., 2009; Hajiboland et al., 2010; Evelin et al., 2012), and ion balance (Giri et al., 2007; Evelin et al., 2012; Wu et al., 2013). For example, plants colonized with *Rhizophagus irregularis* increased the concentrations of nitrogen (N), phosphorous (P), and potassium (K) whereas mycorrhizal plants have decreased (Na) sodium concentrations than the non-mycorrhizal plants at all levels of salinity in tomato and fenugreek plants (Hajiboland et al., 2010; Evelin et al., 2012). Also, plants colonized with *Funneliformis mosseae* maintained higher concentrations of N, P, and K and decreased the Na concentrations in cotton and citrus plants (Wu et al., 2013). Several researchers suggest the role of AM fungi in influencing the ionic balance of the cytoplasm or Na^+^ eﬄux from plants thereby increasing K^+^:Na^+^ ratios under salt stress (Giri et al., 2007; Hajiboland et al., 2010; Evelin et al., 2012; Wu et al., 2013). Moreover, proline content has been reported to change during stress among mycorrhizal plants, and thus, it may serve as a parameter to evaluate the effects of AMF and salinity on plants (Sannazzaro et al., 2007; Echeverria et al., 2013). Several studies have also indicated that AMF symbiosis can increase stomatal conductance, transpiration and photosynthetic rate and water use efficiency in plants exposed to salinity stress than non-mycorrhizal plants (Sheng et al., 2008; Evelin et al., 2012; Ruiz-Lozano et al., 2012; Elhindi et al., 2016; Shamshiri and Fattahi, 2016).

At the same time, variations in mycorrhizal symbiotic efficiency among different plant species have been reported. The mycorrhizal symbiotic efficiency in C_3_ and C_4_ plants varied depending on the plant species involved. Mycorrhizal colonization in C3 plants such as tomato (Abdel Latef and Chaoxing, 2011) and menthol mint (Bharti et al., 2013) were 55 and 64%, respectively. Whereas in C_4_ plants such as maize (Estrada et al., 2013) and *Acacia auriculiformis* (Giri et al., 2003), the mycorrhizal colonization was 64 and 68%, respectively. Moreover, efficiency also varied among isolates of AM fungi irrespective of individual host plant or location of origin. For example, C_3_ plants, *Olea europaea* colonized with *F. mosseae, R. irregularis*, and *R. claroideum*, increased salt tolerance in terms of plant growth and nutrient acquisition particularly N, P, and K uptake. Among AMF species, *F. mosseae* was the most efficient fungus in reducing the detrimental effects of salinity, and this effect was due to increased K uptake (Porras-Soriano et al., 2009). In addition, *Glomus deserticola* exhibited a higher symbiotic efficiency in C_3_ plants, *Lactuca sativa* compared to *Glomus* sp. under saline condition. According to Zou and Wu (2011), the C_4_ plant *Poncirus trifoliate*, trifoliate orange seedlings inoculated with five AMF species (*Diversispora spurca, Claroideoglomus etunicatum, F. mosseae, G. versiforme*, and *Paraglomus occultum*) exhibited different symbiotic efficiency. They proposed that *G. versiforme* showed the best efficiency in alleviating salt stress of trifoliate orange, and *C. etunicatum* exhibited the lowest efficiency. Hence, mycorrhizal development would mostly depend on the compatibility of both AMF and host plants. Enhanced mycorrhizal symbiosis with C_4_ plants, when compared with generally grown C_3_ plants has been documented (Hetrick et al., 1988a; Hetrick, 1991; Wilson and Harnett, 1997). Among C_4_ plants, grasses are reported to be obligate for mycorrhizal symbiosis (Hetrick, 1991). C_4_ grasses showed 98 to 99% mycorrhizal dependency, with positive effects for inoculation resulting up to 63 to 215% greater plant dry weight, than the non-mycorrhizal plants, whereas the C_3_ grasses showed 15 to 75% mycorrhizal dependence and recorded 0.12 to 4.1 times larger plant dry weight (Hetrick et al., 1988b).

The variations in the root morphology of the two plant types play a crucial role in mycorrhizal colonization. C_3_ grasses with fibrous, highly branched root systems are believed to function more independently to support mycorrhizal symbiosis than the C_4_ grass with a coarser and less-branched root system (Hetrick et al., 1990). For woody plants, plant species with thick roots and few root hairs were not much responsive to AMF, whereas woody species with fine roots and abundant root hairs are highly responsive one (Siqueira and Saggin-Junior, 2001). Duponnois and Plenchette (2001) reported a positive correlation on the leguminous mycorrhizal response, the root-hair density and length. It is apparent that the mycorrhizal infection of the root confers less susceptibility to the deleterious effects of salinity and can enable the plants to compensate for root growth and other functions.

Quantitative analytical studies have been widely conducted on AMF colonization and nutrient uptake in normal soils and have also been reported in AMF plant symbiotic relationships under abiotic stress conditions, including drought and metal stresses (Veresoglou et al., 2012; Jayne and Quigley, 2014; Bunn et al., 2015; Pellegrino et al., 2015; Andrade-Linares et al., 2016). Quantifiable validation showed that plants colonized by mycorrhizal fungi have better growth and better reproductive responses under water deficit conditions (Jayne and Quigley, 2014). Despite this fact and despite the in-depth reviews on the AMF mediated salt stress alleviation where data are available (Evelin et al., 2009; Porcel et al., 2012), quantitative analytical studies on these aspects are few. Recently, we conducted meta-analysis report on the symbiotically efficient AMF species and reported that salt-stress alleviation in different plants is due to enhanced nutrient uptake and antioxidant enzyme activity (Chandrasekaran et al., 2014). Additionally, our previous meta-analytical study has confirmed the general overall positive effect in mycorrhizal plants based on plant biomass and nutrient uptake under saline conditions.

Therefore, the present study sought to answer the following questions:

(i) Are there any characteristic differences in plant growth response among C_3_ and C_4_ plants upon mycorrhizal inoculation under salt stress?
(ii) Are there any uptake of specific nutrient (s) essential for salt stresses alleviation in C_3_ and C_4_ mycorrhizal plants?
(iii) Which soil type and salinity level favorably influence the mycorrhizal inoculation in C_3_ and C_4_ plants?
(iv) What are the most effective mycorrhizal inoculation between AMF species and C_3_ and C_4_ plants species under salt stress?

## Materials and Methods

### Literature Search and Data Collection

To build a database, searches were conducted in Web of Science, and the references cited in publications were retrieved from 1998 to 2013. We performed our literature survey using these key words: *C_3_/C_4_ plants^∗^*, ‘*mycorrhiza^∗^/arbuscular mycorrhiza^∗^* and *salt stress/salinity stress^∗^.* Using the Boolean truncation (‘^∗^’) character ensured the presence of words required for a literature survey.

**FIGURE 1:**
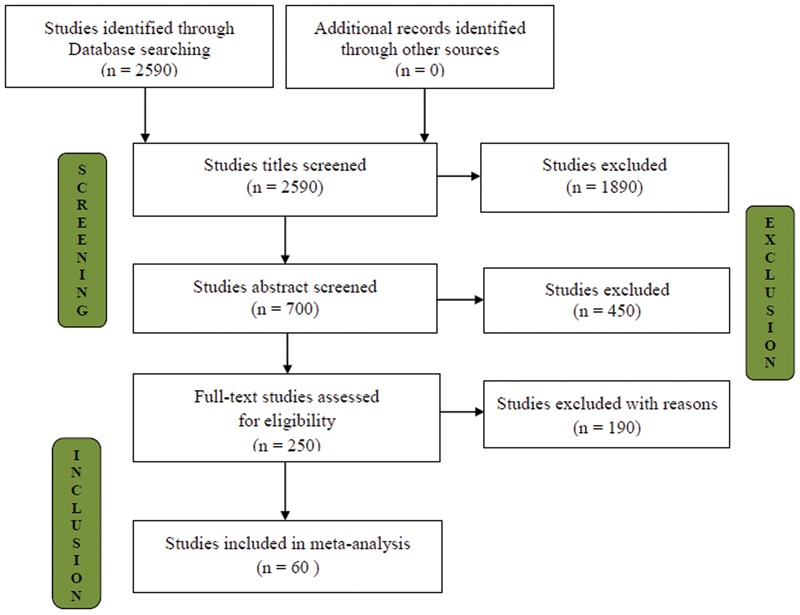
**Selection of studies for inclusion in the meta-analysis**.

These searches resulted in 700 published and unpublished online references. To meet this set of criteria, 250 publications that were likely to contain relevant information were considered (Appendix S1). The selected publications were checked for the following inclusion criteria (i) plant biomass and nutrient uptake, (ii) crop plants (annual and/or perennial), (iii) influence on AMF inoculation compared with uninoculated control and (iv) the experiments that were performed in saline conditions, or at least an EC ≤ 4 dS/m and/or >40 mM NaCl levels. The number of studies selected at various stages is shown in the flow diagram in **Figure 1**. Finally, 60 studies fulfilling all of the required criteria were screened, and 582 trials (bias-corrected) that potentially met the selection criteria of presenting information on the effects of AMF inoculation under salinity stress were selected (see Supplementary Information Dataset). If a particular research paper reported results from more than one study system that had independencies (e.g., AMF inoculum, plant species, and level of salinity), each system was considered as a trial. Based on the criteria mentioned above, our meta-analysis included 229 observations from 25 studies for C_3_ photosynthetic type and 352 observations from 35 studies for C_4_ photosynthetic types.

### Data Acquisition

For each study, we ensured that the data included mean, standard deviation (SD) and replicate number/sample size (*n*) for a control as well as the AMF inoculation under salt stress. If standard errors (SEs) were reported, data were transformed to include SD with the equation *SE* = SD (*n*^-1/2^) using MetaWin 2.1 statistical calculator. In publications where the means and errors were presented in the graph, the image was digitized, and Dexter (GAVO data center) software was used to estimate the values^1^.

### Effect Size and Publication Bias

Effect sizes were calculated as the natural log of response ratio (LRR) as a metric for the response of AMF inoculation under salinity stress, due to its statistical properties (approximate normal distribution) and biological interpretation (Hedges et al., 1999; Rosenberg et al., 2000). These calculations were conducted by the Meta-Win v2.1 software (Rosenberg et al., 2000).

$$\begin{array}{c} LRR\;=\;ln\;\left(\frac{{\bar{X}}^{E}}{{\bar{X}}^{C}}\right)=\;ln\;\left({\bar{X}}^{E}\right)-ln\left({\bar{X}}^{C}\right)\;\\ V_{\ln\;R}=\frac{{\left(S^{E}\right)}^{2}}{N^{E}{\left({\bar{X}}^{E}\right)}^{2}}+\frac{{\left(S^{C}\right)}^{2}}{N^{C}{\left({\bar{X}}^{C}\right)}^{2}}\; \end{array}$$

where *R* is the response ratio, *X*^C^ is the control mean (without AMF inoculation under salt stress), *X*^E^ is the treatment mean (with AMF inoculation under salt stress), *S*^C^ is the control SD, *S*^E^ is the treatment SD, *N*^C^ is the control replication number, and *N*^E^ is the treatment replication number.

In the framework of our meta-analysis, under salinity stress, each response parameter observed after AMF inoculation was assessed by Spearman’s rho rank correlating effect size against sample size. If larger effect sizes were more likely to be published than smaller effect sizes, a significant correlation would indicate publication bias. We utilized the fail-safe number of the Rosenthal method to determine the number of non-significant, unpublished or missing studies, which would have to be added to our meta-analysis to nullify its overall effect size (Rosenberg, 2005). If this number was larger than 5*n* + 10 (*n* = number of studies), then publication bias was safely ignored. We also checked the existence of publication bias via scatter-plots and/or funnel plots of effect size vs. sample size or variance, respectively, according to Nakagawa and Santos (2012; Supplementary Figures S1 and S2).

### Categorical Independent Variables

Categorical analyses were made on the data to determine the comparative responses of C_3_ and C_4_ plants to AMF inoculation under soil salinity by considering the different fixed factors mentioned below.

*Photosynthetic type* had two levels: C_3_ and C_4_ plants corresponding to the two data sets, which allowed testing for any significant differences on the AMF-mediated plant growth and nutrient uptake under salt stress among photosynthetic types.

*Plant group* had two levels: *monocot* and *dicot.* The plants were classified using the PLANTS database of the USDA, Natural Resources Conservation Service^2^.

*Plant types*: Studies were coded to include the following variables: *annual herb, perennial herb, woody*, and *grass.*

*Plant growth habit* had seven levels: *forb/herb, shrub, herb, grass, tree, sub shrub*, or *shrub/tree*.

*Soil type* had five levels: *sandy, sandy loamy, loamy, clayey loamy*, and *silty* soil. Soil types were classified using the soil database of the USDA Natural Resources Conservation Service^2^.

*Soil salinity* had three levels: *low, moderate*, and *high* salinity. The levels of salinity data reported were classified, referring to the USDA Natural Resources Conservation Service. Accordingly, *Low level salinity* with EC ≤ 4 dS/m, *moderate level salinity* with 4–8 dS/m and *high salinity* with more than 8 dS/m were considered.

*AMF inocula* had two levels: *single* and *mixed*. Single species inoculants were considered using one of the AMF species, whereas the mixed species inoculants had more than one AMF species. The AMF inoculants used were either isolated from field soil or obtained from commercial suppliers. The AM fungal species were classified and named according to the nomenclature of Redecker et al. (2013).

*Plant species and plant family*: Forty plant species belonging to the families of Anacardiaceae, Asteraceae, Caryophyllaceae, Chenopodiaceae, Euphorbiaceae, Fabaceae, Lamiaceae, Liliaceae, Malvaceae, Moraceae, Poaceae, Rutaceae, Solanaceae, and Verbenaceae were included in our analysis.

*Experimental duration* had three levels: short studies lasting up to 2 months; intermediate studies of 2–4 months and long-time studies of more than 4 months were considered.

*Experimental condition* had two levels: *greenhouse experiments* conducted indoors under controlled environmental conditions and *field experiments* conducted in uncontrolled environmental conditions were considered.

### Statistics

Random-effects model meta-analyses were conducted for each of the categorical independent variables to test their influence on the impact of AMF inoculation and the alleviation of salt stress effects in C_3_ and C_4_ plants using MetaWin 2.1 software. All models were weighted by the inverse of variance in LRR (Rosenberg et al., 2000). A permutation procedure with 4,999 iterations was used because the number of their effects violated the criterion of normality (Adams et al., 1997). Confidence intervals were then estimated through a bootstrap procedure that implemented bias-correction (Rosenberg et al., 2000).

Most of the studies included in the datasets contained more than one trial in experimental setup. To handle this common issue in ecological meta-analyses and the independence violation of these studies (Nakagawa and Santos, 2012), the following two modifications were applied: (i) if multiple trials shared the same control, they were modified using the methodology of Lajeunesse (2011), and (ii) multiple trials originating from the same study were brought to a single effect size through fixed-effects using the meta-analytical procedure (Lajeunesse, 2011; Lehmann et al., 2014). These approaches ensured that the random-effects component of the meta-analysis was restricted to trials that belonged to different studies. Trials of one study were not reduced if the effect sizes that originated from different experimental systems were represented by independent variables (Rosenberg et al., 2000).

Potentially meaningful statistical inferences are presented for those variables for which data for at least two categories were available for either C_3_ or C_4_ datasets containing reasonable sample sizes. This was done to allow at least a rudimentary comparison between C_3_ and C_4_ species. For each categorical analysis, the total heterogeneity was calculated among studies (*Q*_T_) within group heterogeneity (*Q*_W_), or between group heterogeneity (*Q*_B_). Studies were considered significant when *Q*_B_ was significant (*P* < 0.05) and described at least 10% of the total variation (*Q*_B_/*Q*_T_ ≥ 0.1; Rosenberg et al., 2000). Zero (0) effect sizes signify no difference in effects between the experimental and control groups, negative values represent effects where the control group attains a greater significance than the experimental group, and positive values represent effects where the experimental group attains a greater significance than the control group. AMF inoculation effects were estimated as a percentage change, relative to the control (%), using the equation [exp (LRR) - 1] × 100. A sensitivity analysis was conducted to test for any disproportional impact of single studies and their reproducibility (Hedges et al., 1999). In this study, we tested significant results, and only robust or bias-corrected results are presented in the results section (Supplementary Figures S3–S5).

## Results

### Overall AMF Inoculation Effects on Plant Growth and Nutrient Uptake under Salt Stress

The results of meta-analysis of 60 studies conducted on different host plants under salt stress show that mycorrhizae are a significant factor affecting plant growth and nutrient uptake in both C_3_ and C_4_ plants irrespective of plant photosynthetic type (Supplementary Figure S6; Supplementary Table S1). Shoot, root and total biomass of AMF-inoculated plants increased by 67.1, 57.8, and 71.1%, respectively. Also, the AMF-inoculated plants under saline conditions significantly increased N, P, and K uptake by 93.2, 86.8, and 42.7%, respectively. In addition, Na uptake and proline accumulation decreased significantly by 22.2 and 6.5%, respectively, under saline condition in AMF inoculated plants (Supplementary Figure S6; Supplementary Table S1).

Under normal condition our analysis provided strong, quantitative evidence that AM fungi positively influenced plant biomass (shoot, root and total biomass by 32.3, 24.8, and 39.3%, respectively) and nutrient uptake (N, P, and K uptake by 17.1, 37.5, and 21.5%, respectively) in both C_3_ and C_4_ plants irrespective of photosynthetic types. In addition, overall AMF inoculation responses increased positively in both C_3_ and C_4_ plants by 23.7 and 25.3%, respectively. Furthermore, both C_3_ and C_4_ plants increased total biomass by 31.8 and 46.1%, respectively, relative to uninoculated controls. C_3_ plants showed greater AMF mediated increase in P uptake (35.6%) and N uptake (19.9%) compared to C_4_ plants P uptake (33.5%) and N uptake (16.7%), however, no statistical differences were observed between C_3_ and C_4_ plants. We also found that AMF inoculation increased K uptake by 21.7 and 22.8% for C_3_ and C_4_ plants, respectively. On the other hand, Na uptake decreased by 2.1% in C_3_ plants and increased by 23.6% in C_4_ plants. Mycorrhizal inoculation increased proline accumulation in C_4_ plants by 19.1% and C_3_ plants by 5.6% compared to noninoculated plants (Supplementary Figure S7).

### C_3_ vs. C_4_ Plant Response to AMF Inoculation in Saline Soils

**FIGURE 2:**
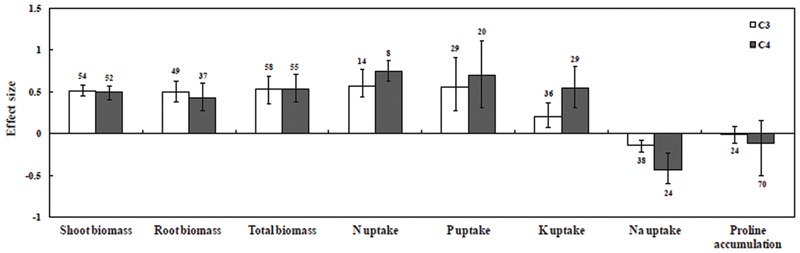
**Comparative photosynthetic growth and nutrient uptake responses of arbuscular mycorrhizal fungi (AMF)-inoculated plants under salt stress.** Error bars are means ± bias CIs. Where the CIs do not overlap the horizontal dashed lines, the effect size for a parameter is significant at *P* < 0.05. A number of studies were included for the meta-analysis mentioned above the bar.

Results illustrated that overall AMF inoculation responses increased in both C_3_ and C_4_ plants by 42.9 and 44.5%, respectively (**Figure 2**; Supplementary Table S2). Both C_3_ and C_4_ plants increased total biomass under saline conditions by 69.5 and 70.8%, respectively, relative to uninoculated controls. Mycorrhizal inoculation was also found to increase shoot biomass and root biomass in C_3_ and C_4_ plants under saline conditions. In addition, AMF inoculation mediated increase in P uptake was observed in both C_3_ (73.1%) and C_4_ plants (101.4%). In C_3_ and C_4_ plants, N uptake increased by 77.6 and 112.3%, respectively, but due to low sample size this result needs to be treated with caution. K uptake in C_4_ plants increased significantly (*P* ≤ 0.05) compared to C_3_ plants. Na uptake decreased significantly by 13.4% in C_3_ plants and 35.2% in C_4_ plants. On the other hand, C_4_ plants showed a 10.9% decrease in proline accumulation and C_3_ plants showed a 1.6% decrease.

### C_3_ and C_4_ Plant Response to Plant Types and Fungal Symbionts

**FIGURE 3:**
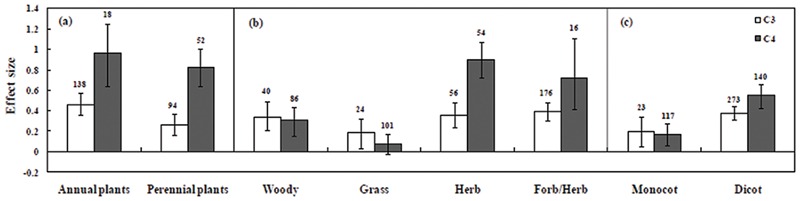
**Arbuscular mycorrhizal fungi inoculation responses of plants under salt stress.** Categorical analysis of **(A)** plant duration, **(B)** plant functional groups, and **(C)** plant types. Error bars are means ± bias CIs. Where the bias CIs do not overlap the horizontal, dashed lines, the effect size for a parameter is significant at *P* < 0.05. All effect sizes differed significantly from zero. The number of studies is shown above the bar.

The categorical analysis of plant types, plant groups and growth habitat had a significant effect on effect sizes (**Figure 3**). Among C_3_ mycorrhizal plants, *Cicer arietinum* followed by *L. sativa, Lotus glaber, Cajanus cajan*, and *Solanum lycopersicum* had significant positive effect size. C_4_ species *A. nilotica, Cyamopsis tetragonoloba, Gmelina arborea, A. auriculiformis*, and *Allium sativum* were found to be effective under salt stress. Moreover, C_3_ plants such as *L. tenuis* had significant negative effect size on plant growth. Among C_4_ plants *P. trifoliate and Spartina alterniflora* and some trials with *Zea mays* had negative responses under saline conditions. The significant positive effect size on plant types under salt stress was recorded on both monocotyledonous and dicotyledonous plant types (**Table 1**). In the analyses of data indicated in C_3_ plants, monocotyledonous plants showed increased effect size whereas in C_4_ plants, dicotyledonous plants showed increased effect size under salt stress conditions compared to those of monocotyledonous type (**Figure 3**). Plant type (annual vs. perennial) had a significant (*P* < 0.05) effect size as evidenced by the result that C_4_ perennial plants have higher effect size, than C_3_ plants. In our study we have also observed that C_4_ annual plants performed better than C_3_ plants under mycorrhizal condition. No significant effect size was observed in C_4_ grass, in which the confidence interval overlapped with zero. Among plant functional groups, mycorrhizal C_4_ herbs were found to have more effective amelioration of salt stress compared with other functional groups (**Figure 3**).

**Table 1:** Comparative photosynthetic types significance analyzed in the categorical analyses based on the significance of the variation among categories (*Q*_B_) and the amount of the total variation (*Q*_T_) described by *Q*_B_/*Q*_T_ under salt stress.

Categorical independent variable		*Q*_B_	*Q*_B_/*Q*_T_	*P*_random_
Response variable	C_3_	229.8693	0.2172	0.0002
	C_4_	116.8027	0.2625	0.0002
AMF species	C_3_	16.7240	0.0238	0.2232
	C_4_	96.9317	0.1881	0.0004
AMF inoculation	C_3_	12.7338	0.0173	0.0278
	C_4_	0.7907	0.003	0.4666
Plant species	C_3_	222.275	0.2735	0.0002
	C_4_	217.6239	0.3431	0.0002
Plant group	C_3_	57.8540	0.173	0.0036
	C_4_	137.3704	0.2376	0.0002
Plant family	C_3_	41.2789	0.1340	0.0420
	C_4_	104.0544	0.1858	0.0002
Plant types	C_3_	56.0280	0.0937	0.0002
	C_4_	88.2995	0.1595	0.0002
Plant growth habit	C_3_	24.5270	0.0351	0.0174
	C_4_	135.757	0.2359	0.0002
Experimental condition	C_3_	1.0427	0.0014	0.5044
	C_4_	0.1821	0.00001	0.7876
Level of salinity	C_3_	61.6394	0.1190	0.0716
	C_4_	155.1245	0.2393	0.0002
Degree of salinity	C_3_	1.5496	0.0021	0.7272
	C_4_	16.0778	0.0374	0.0050
Experimental duration	C_3_	24.0317	0.0239	0.0084
	C_4_	33.999	0.1604	0.0006
Soil type	C_3_	32.1172	0.1552	0.0420
	C_4_	25.4710	0.10043	0.0070

**FIGURE 4:**
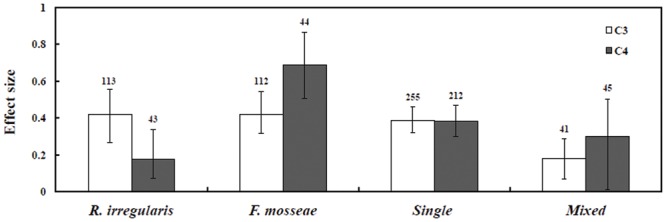
**Effect sizes of AMF species-categorical analysis.** Error bars are means bias CIs. Where the CIs do not overlap the horizontal dashed lines, the effect size for the parameter is significant at *P* < 0.05. A number of studies included for meta-analysis are mentioned above the bar.

Different AM fungal inoculation types increased the growth of both plant types under saline conditions. Single and mixed AMF species inoculations differed significantly. C_3_ plants showed increased effect size in single species inoculation compared with C_4_ plants. On the other hand, C_4_ plants showed a greater increase in effect size with mixed AMF inoculation than the C_3_ plants (**Figure 4**). Categorical analysis indicated significant (*P* = 0.0002) differences among AMF species (**Table 1**). Among the AMF species, *R. irregularis* and *F. mosseae* showed increase in effect size of C_3_ species by 52.3 and 52.3%, respectively, compared with C_4_ plants with a 19.2 and a 99.1% increase, respectively. Nevertheless, *R. irregularis* alone had a significant difference for C_3_ and C_4_ plants under salinity stress.

### C_3_ and C_4_ Plant Response to Soil Texture, Salinity and Experimental Settings

**FIGURE 5:**
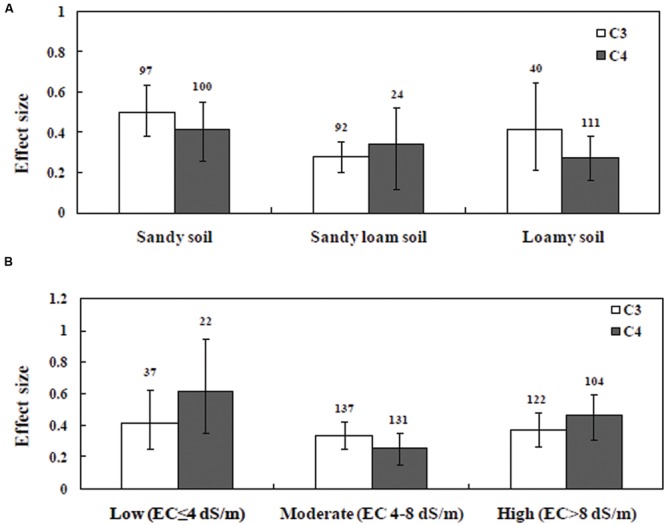
**Arbuscular mycorrhizal fungi inoculation responses of plant under salt stress.** Categorical analysis of **(A)** soil types **(B)** degree of salinity. Error bars are means ± bias CIs. Where the bias CIs do not overlap the horizontal dashed lines, the effect size for a parameter is significant at *P* < 0.05. The number of studies included for the meta-analysis is mentioned above the bar.

Soil type significantly influenced the effect sizes of C_3_ and C_4_ datasets (**Figure 5A**). In the C_3_ dataset, plants grown in sandy soil yielded higher effect size than plants grown in silty or clay loamy soil. On the other hand, in the C_4_ dataset, silty soil yielded higher effect size than sandy soil. In addition, the effect size of C_3_ plants was significantly increased due to mycorrhizal inoculation than it was in C_4_ plants grown under salinity stress. The effect of soil salinity on the relative response to AMF inoculation is presented in **Figure 5B**. Soil salinity significantly influenced the effect size in plants (*P* < 0.05). AMF-inoculated plants grown in moderate salinity showed a significant difference between C_3_ and C_4_ plants, whereas effect in size variation between C_3_ and C_4_ plants under low and high salinity levels were not significant. AMF-inoculated plants grown in moderate salinity yielded higher effect size for C_3_ plants compared with those of C_4_ plants (**Figure 5B**).

In our dataset, experimental duration significantly influenced the effect size in both C_3_ and C_4_ plants. AMF inoculation in short-duration C_4_ plants had a significant effect on plant growth compared with that of C_3_ plants. On the other hand, C_3_ species showed a greater increase in effect size of intermediate duration (**Table 1**). Experimental conditions had a significant effect on the C_3_ dataset where plants grown under controlled-greenhouse conditions had higher effect size values compared to that of the C_4_ dataset (**Table 1**). However, C_4_ plants showed a greater increase in effect size under field conditions when compared to the C_3_ plants.

### C_3_ and C_4_ Plant Response to Biomass

Among plant biomass studies, AMF inoculation had a significant positive effect (67.11%, *P* < 0.05) on shoot biomass whereas no significant (*P* > 0.05) effects were detected in root and total biomass under saline condition (**Figure 2**; Supplementary Table S2). Categorical analysis of shoot biomass showed that among AMF species, *R. irregularis* followed by *F. mosseae* were found to be effective with C_3_ plants. On the other hand *F. mosseae*, followed by *R. fasciculatus* and *R. irregularis* were effective with C_4_ plants.

### C_3_ and C_4_ Plant Response to P, N, and K Uptake

Among all nutrient studies included for meta-analysis, P uptake (86.8%) showed a significant positive response for all types of categorically fixed factors. A significant effect on P uptake was found in this study (Supplementary Figure S6; Supplementary Table S2). Although Kendall’s tau and Spearman rank correlation test for publication bias was found to be significant, this was ignored based on Rosenthal Fail-safe calculation (Supplementary Table S2). Categorical analysis of P uptake among AMF species was found to have a significant positive effect under salt stress (*P* = 0.02). Among AMF species, C_4_ plants inoculated with *R. fasciculatus* had a more significant positive effect than *R. irregularis* and *F. mosseae*, whereas an opposite trend was observed in C_3_ species. Across the study, single inoculants recorded higher P uptake than mixed inoculants in both C_3_ and C_4_ species (*P* > 0.05). In addition, there were significant differences among plant species for P uptake under salt stress (*P* = 0.01). Among C_4_ plant species, *A. nilotica* and *C. tetragonoloba* were found to be efficient for P uptake, whereas among C_3_ plant species, *Trifolium subterraneum, C. arietinum*, and *S. lycopersicum* had larger effect size. The significant positive effects of AMF inoculation on P uptake under salt stress were recorded, and there were higher levels with dicotyledonous plants than with monocotyledonous plants. Among the plant photosynthetic types, though, P uptake had a positive effect on size; their effect on mycorrhizal inoculation did not differ between C_3_ and C_4_ plants (**Figure 2**; Supplementary Table S2). Furthermore, categorical analyses indicated that there was no significant difference between the effect sizes for P uptake in plants with response to varied degrees of salinity. However, at a moderate degree of salinity, P uptake was higher when compared with those at high and low levels of salinity. There was no significant difference observed between experimental duration trials. Short-duration studies showed a higher effect size in P uptake compared with moderate- and long-duration studies under salt stress.

In this study, it was observed that the N uptake (93.2%) had a positive effect size for AMF inoculation under salt stress conditions (Supplementary Figure S6; Supplementary Table S2). Significant publication bias observed in Kendall’s tau and the Spearman rank correlation was safely ignored (Supplementary Table S2). Categorical analysis among groups was not significant, except for plant functional groups and plant species, indicating inconsistencies and low sample sizes in the mycorrhizal plants in response to N uptake under salt stress. Among plant photosynthetic types, N uptake increased positively in C_3_ and C_4_ plants, but C_4_ plants had increased effect size (**Figure 2**).

K uptake showed variation among studies, but it was consistently significant in AMF inoculated C_3_ and C_4_ plants (Supplementary Figures S6 and S8; Supplementary Table S2). Categorical analyses showed significant differences between AMF species. In C_4_ plants, *R. fasciculatus* showed a greater increase in K uptake than *F. mosseae* and *R. irregularis*. However, in C_3_ species, *F. mosseae* followed by *R. irregularis* was found to be effective. Categorical analysis of plant species also showed a significant positive effect (*P* = 0.0002) on K uptake under saline conditions. K uptake in C_4_ plants was found to be significantly greater than those of C_3_ plants (**Figure 2**). Among C_4_ species, increased K uptake was observed in *A. nilotica* (due to low sample size, treat the results with caution) followed by *Z. mays*, while in C_3_ species, *S. lycopersicum* was found to be more effective for K uptake. When compared among all plant functional groups (*P* = 0.0002), K uptake in herbs was significantly greater than those of tree and grass types. In addition, the K uptake in a low and high degree of salinity was significantly higher than moderate salinity across studies.

### C_3_ and C_4_ Plant Response to Na Uptake and Proline Accumulation

In contrast to N, P, and K uptake, mycorrhizal plants had a consistent negative response to Na uptake by an average of 22.2% across all studies. Furthermore, variations among studies were significant (*P* < 0.05, Supplementary Figure S6; Supplementary Table S2), indicating consistency among groups with mycorrhizal responses to salt stress. Among AMF species, *R. irregularis* was found to have a negative impact on C_4_ plants. On the other hand, *F. mosseae* showed a negative effect size in C_3_ plants under salt stress. Categorical analysis indicates that plant species (*P* = 0.001) and plant duration (*P* = 0.02) contributed significantly to Na uptake. Among the plant types, woody plants were found to have more negative impact for Na uptake, but bias CI overlapped with zero. Grasses were significantly negative for Na uptake in AMF inoculated plants. Among plant photosynthetic type, C_4_ plants were found to be more negative for Na uptake than those of C_3_ plants. Among plant species, Na uptake for *S. lycopersicum* was found to be more negative in C_3_ plants, whereas *Z. mays* had a more negative size in C_4_ plants. Moreover, a negative effect for Na uptake was comparatively greater in monocotyledonous plants than in dicotyledonous plants.

Both increases and decreases in proline accumulation under salt stress have been ascribed to AM symbiosis. The present study showed that proline accumulation has been found to decrease when the plant is colonized by AMF (6.5%), but this effect was not significant since the confidence intervals overlapped with zero (Supplementary Figure S6; Supplementary Table S2). However, categorical analysis of AMF species showed that in both C_3_ and C_4_ plants, *R. irregularis* and *F. mosseae* inoculated plants had more reduced level of proline accumulation but, *R. fasciculatus* inoculated plants had increased proline accumulation under salt stress. Within the categorical analysis of C_3_ mycorrhizal plants, increased proline content compared to non-mycorrhizal plants under saline condition was observed in *M. arvensis, C. arietinum*, and *L. glaber* plants. However, within C_4_ mycorrhizal plants under saline condition, significant positive effect on proline accumulation was seen in *G. arborea, Jatropha curcas*, and *A. sativum* plants, but *Z. mays* and *Pennisetum glaucum* showed significant negative effects on proline accumulation. Among plant functional group, C_4_ monocotyledonous plants had reduced level of proline accumulation, whereas C_3_ dicotyledonous plants had significant increase in proline accumulation than those of non-mycorrhizal plants. All of the other plant functional groups and experimental conditions showed no significant differences in proline accumulation under salt stress.

## Discussion

### AMF Symbiotic Efficiency of C_3_ and C_4_ Plants in Saline Soils

Plant growth diminishes under salt stress due to (a) spending of more energy to avoid the toxic effects of NaCl and (b) deficiency in nutrients (Munns and Tester, 2008). On the other hand, mycorrhizal inoculation was found to enhance the efficiency of the host plants by increasing their growth and photosynthetic efficiency (Chandrasekaran et al., 2014; Elhindi et al., 2016; Shamshiri and Fattahi, 2016). In the present study, we have observed that the overall AMF inoculation response increased in both C_3_ and C_4_ plants, and the total plant biomass was found to be enhanced in both C_3_ and C_4_ plants. Similar results were also observed for both C_3_ and C_4_ plants under normal condition. Previous meta-analyses have also documented enhanced plant growth due to AMF inoculation (Hoeksema et al., 2010; Treseder, 2013; Pellegrino et al., 2015). Resolute evidence was also provided for AMF species causing a significant impact on many ecological predictor variables on C_3_ and C_4_ photosynthetic plant types under salinity stress. Thus far, studies on salt-stress tolerance in mycorrhizal plants have suggested that AMF inoculated plants grew better due to improved mineral nutrition and physiological processes such as photosynthesis or water use efficiency, production of osmoregulators, higher K^+^/Na^+^ ratios and compartmentalization of sodium within some plant tissues (Giri et al., 2007; Elhindi et al., 2016). The saline-stress-alleviation effect on C_3_ and C_4_ plants may be attributed to enhanced mycorrhizal growth response. In a saline environment, root growth is delayed with the effect of salt on cell toxicity and due to low soil water potential (Psarras et al., 2008). An increase in salt concentrations proportionately increases the mycorrhizal response in *Sesbania* and *Gmelina* plants (Giri and Mukerji, 2004; Dudhane et al., 2011). We also observed salinity-level variations in the mycorrhizal symbiosis of C_3_ and C_4_ plants. AMF-inoculated plants grown in moderate salinity yielded a higher effect size for C_3_ plants than those of C_4_ plants.

### Nutrient (s) Mediated Salt Stress Tolerance in C_3_ and C_4_ Mycorrhizal Plants

Overall meta-analysis showed a significant (*P* < 0.05) increase in mean-effect sizes in mycorrhizal plants in all salinity levels, indicating the fact that AMF inoculation increased P uptake. The ability of AM fungal to acquire P is known to differ among isolates, and a similar result is expected for other functions as well (Hoeksema et al., 2010; Treseder, 2013). C_4_ plants inoculated with *R. fasciculatus* showed a more positive effect than *R. irregularis*, and *F. mosseae*, whereas the opposite trend was observed in C_3_ species. Therefore, our results suggest that environment (i.e., the level of salinity) restricted an association to a single species. A higher response to P uptake was observed in the Fabaceae family for both C_3_ and C_4_ plants, indicating many trends common to both photosynthetic types. In ways similar to our study, Treseder (2013) provided meta-analytical evidence for a positive, significant relationship between per cent root colonization and plant biomass due to high P uptake.

In our analysis, we found that among plant photosynthetic types, mycorrhizal C_4_ plants had a higher N uptake than C_3_ plants, but due to low sample size this result needs to be treated with caution. This could be explained based on the C_4_ mode of photosynthesis that had indirect consequences on the use of water by these plants (Ehleringer and Monson, 1993). Improved efficiency in nitrogen use during photosynthesis has been suggested to allow C_4_ plants to develop a higher leaf area for effective carboxylation and the translocation of photosynthates toward roots under nitrogen-limited conditions, and this favored the increase in mycorrhizal colonization for nutrient acquisition. However, to the best of our knowledge, the exact mechanism for why AMF enhances N nutrition under salt-stress conditions is yet to be understood.

K plays a number of essential roles in plants, including enzyme activation, protein synthesis, photosynthesis, osmoregulation, stomatal regulation, energy transfer, phloem transport, cation-anion balance, and stress resistance (Wang et al., 2013). Salinity stress decreases the level of K^+^ as a competitor of Na^+^. Because, Na^+^ and K^+^ have similar physiological properties, cytoplasmic Na^+^ fights for similar binding sites and hence limits the metabolic processes that depend on K^+^ (Shabala and Cuin, 2008). Mycorrhizal inoculation can enhance the K absorption under saline conditions and prevent the translocation of Na. Thus, increasing the K^+^/Na^+^ ratio by the AMF inoculation under high salinity levels could be one of the reasons for enhancing plant tolerance to the highest salinity level through Na exclusion (Tang et al., 2006; Giri et al., 2007; Elhindi et al., 2016). In the current study, we have seen that K uptake by C_3_ and C_4_ plants were positively influenced by the AMF inoculation, and K uptake was significantly higher in C_4_ plants. Salt-stressed root growth is restricted by the osmotic and toxic effects of ions, which result in lower nutrient uptake and inhibition of the translocation of mineral nutrients, especially K^+^ (Wang et al., 2013). Therefore, we suggest that the inoculation enhancing exploration of the external mycorrhizal hyphae beyond the nutrient-depleted zones can increase P and K uptake in both plant types.

### C_3_ and C_4_ Plant Species Responding to AMF Symbiosis

The present study showed that C_4_ plants had more significant increase in P uptake efficiency compared with C_3_ plants under salt stress. This result may be attributed to the fact that C_4_ plants seem to be highly benefited from mycorrhizal inoculation than the selected C_3_ plants due to the preferential P allocation for photosynthesis in those C_4_ plants (Tang et al., 2006). Under high-salinity stress, enhanced *de novo* biosynthesis of CO_2_ concentrating phosphoenolpyruvate carboxylase (PEPC) enzyme in C_4_ plants requires more P for their additional ATP requirement (Doubnerova and Ryslava, 2011). AMF inoculation can help plants meet out their P demand for improving their survival with salt stress (Doubnerova and Ryslava, 2011). The retention of P within tissues of C_4_ plants could elicit higher total P content within the plant (Kishor et al., 2005). Therefore, even a little increase in nutrient uptake via AMF may contribute to a larger increase in plant P content (Maherali and Klironomos, 2012).

The present analysis reveals that C_4_ plant species showed significantly better response to mycorrhizal inoculation particularly for K uptake. Among C_4_ plants, *Z. mays* was found to be more competitive for K^+^ uptake. Automatic exclusion of Na^+^ ions in the soil could be achieved by C_4_ plants in saline soils (Omoto et al., 2012). This was again reflected by a decrease in proline accumulation in C_4_ compared to C_3_ plants under salinity stress (Omoto et al., 2012). AMF inoculated plants showed a significantly higher K and lower Na uptake than the uninoculated ones under salinity stress, which suggests a preferential intake of K^+^ rather than of Na^+^ into the root xylem. Therefore, the enhancement of growth in mycorrhizal plants under saline conditions could be attributed to decreased Na^+^ uptake, with enhanced K uptake resulting in a higher K^+^/Na^+^ ratio (Elhindi et al., 2016).

The investigations carried out so far on proline accumulation in AMF symbiosis are limited, and contradictory (Kishor et al., 2005). Proline accumulation was found to increase when the plant was colonized with AMF (Maiale et al., 2004; Echeverria et al., 2013). Some studies demonstrated that AMF inoculation significantly decreased proline accumulation (Jahromi et al., 2008; Borde et al., 2011), and several other studies also showed little or no effect on proline accumulation (Hajiboland et al., 2010). Across the studies, we could find variations in proline accumulation among *Glomus* spp. The inoculation of *R. fasciculatus* and *Glomus* sp. increased proline accumulation, whereas a decrease in proline accumulation was observed in *R. irregularis* and *F. mosseae* inoculated C_3_ and C_4_ plants. In addition, C_3_ plant species (*M. arvensis* and *C. arietinum*) and some of the C_4_ plant species (*G. arborea, J. curca*, and *A. sativum*) showed a significant increase in proline accumulation, whereas some of the C_4_ plant species (*Z. mays* and *P. glaucum* and) and C_3_ plant species (*Trigonella foenum* and *S. lycopersicum*) had a significant decrease in proline accumulation. The low accumulation of proline in AMF-inoculated C_4_ plants may be attributed to their inherent higher salinity stress tolerance than the high-proline accumulation in stressed C_3_ plants. An increase or a decrease in plant proline was also found to be directly proportional to Na uptake; as decreased Na uptake in C_4_ plants decreased proline accumulation. In addition to this, mycorrhizal inoculation had no effect on proline accumulation in C_3_ plants. Earlier research studies also support the present quantitative analytical finding that the proline accumulation response to salt stress is a good indicator of stress perception among plant species with regards to their saline tolerance ability (Evelin et al., 2009; Garg and Chandel, 2012).

## Conclusion

With quantitative analytical evidence, we suggest that AMF inoculation positively influences plant growth and nutrient uptake in both C_3_ and C_4_ plant types in saline soils. Among AMF species, *F*. *mosseae* was found to be well suited for C_3_ plants, whereas *G*. *fasciculatum* showed a high growth response with the C_4_ plants under salinity stress. Mycorrhizal inoculation in both C_3_ and C_4_ plants under salinity stress exhibited an intriguing pattern of responses, where higher effectiveness of AMF inoculation was observed under low and high salinity levels than under moderate levels. But, significantly high symbiotic efficiency was observed in C_3_ mycorrhizal plants under moderate levels of salinity. Our study also described that under mycorrhizal conditions, C_3_ annual plants performed significantly higher than C_4_ plants, with an exception in perennial plants. Future research on mycorrhizal symbioses with C_3_ perennial plants and C_4_ annual plants can answer several questions that arose from the present analysis and may lead to progress in symbiotic efficiency of mycorrhizal fungi under salinity stress. Among the two plant photosynthetic types, C_4_ plants showed more competitive K^+^ ion uptake than C_3_ plants. Salt-stressed, AMF-inoculated C_4_ plants exhibited higher K^+^/Na^+^ ratio than those of salt stressed C_3_ plants and non-mycorrhizal plants. Studies on the detailed mechanism for the selective transport of K in C_3_ and C_4_ mycorrhizal plants under salt stress are lacking, and this needs to be explored. Our study concludes that AMF inoculation had a positive effect on plant growth and nutrient uptake in both C_3_ and C_4_ plants grown under salinity stress contrary to current perceptions.

## Author Contributions

MC and TS: Conception and design of the work. MC: Performed the work. MC, KK, RK, and DW: acquisition of data. MC, SS, SH, S-HO, and TS: analyzed the data. MC, KK, RK, SS, SH, MJ, S-HO, GS, and TS: critical revision of manuscript. MC, MJ, GS, and TS: wrote the paper.

## Conflict of Interest Statement

The authors declare that the research was conducted in the absence of any commercial or financial relationships that could be construed as a potential conflict of interest.

## Footnotes

1 http://dc.zah.uni-heidelberg.de/sdexter/

2 http://plants.usda.gov/java/
