# Supplementary material for: Mycorrhizal Symbiotic Efficiency on C3 and C4 Plants under Salinity Stress – A Meta-Analysis
Source: Front Microbiol. 2016 Aug 11;7:1246. doi: 10.3389/fmicb.2016.01246 (PMC4981042; doi:10.3389/fmicb.2016.01246)
Supplement: Supplementary file 1 [file Data_Sheet_1.DOCX]

**Appendix S1.** Study references of the studies included in the meta-analysis.

**References included in the meta-analysis**

1. Abdel Latef A.A.H., and Chaoxing, H. (2011). Effect of arbuscular mycorrhizal fungi on growth, mineral nutrition, antioxidant enzymes activity and fruit yield of tomato grown under salinity stress. *Scientia Horticulturae* 127, 228–233.
2. [Aroca, R](http://www.ncbi.nlm.nih.gov/pubmed?term=Aroca%20R%5BAuthor%5D&cauthor=true&cauthor_uid=23102876)., [Ruiz-Lozano, J.M](http://www.ncbi.nlm.nih.gov/pubmed?term=Ruiz-Lozano%20JM%5BAuthor%5D&cauthor=true&cauthor_uid=23102876)., [Zamarreño, A.M](http://www.ncbi.nlm.nih.gov/pubmed?term=Zamarre%C3%B1o%20AM%5BAuthor%5D&cauthor=true&cauthor_uid=23102876)., [Paz, J.A](http://www.ncbi.nlm.nih.gov/pubmed?term=Paz%20JA%5BAuthor%5D&cauthor=true&cauthor_uid=23102876)., [García-Mina, J.M](http://www.ncbi.nlm.nih.gov/pubmed?term=Garc%C3%ADa-Mina%20JM%5BAuthor%5D&cauthor=true&cauthor_uid=23102876)., [Pozo, M.J](http://www.ncbi.nlm.nih.gov/pubmed?term=Pozo%20MJ%5BAuthor%5D&cauthor=true&cauthor_uid=23102876)., and [López-Ráez, J.A](http://www.ncbi.nlm.nih.gov/pubmed?term=L%C3%B3pez-R%C3%A1ez%20JA%5BAuthor%5D&cauthor=true&cauthor_uid=23102876). (2013). Arbuscular mycorrhizal symbiosis influences strigolactone production under salinity and alleviates salt stress in lettuce plants. [*J. Plant Physiol*](http://www.sciencedirect.com/science/journal/01761617). 170, 47−55.
3. Asghari, H.R. (2004). Effects of Arbuscular mycorrhizal fungal colonization on management of saline lands. The University of Adelaide. <http://digital.library.adelaide.edu.au/dspace/bitstream/2440/37965/2/01Front.pdf>.
4. Asghari, H.R., Marschner, P., Smith, S.E., and Smith, F.A. (2005). Growth response of *Atriplex nummularia* to inoculation with Arbuscular mycorrhizal fungi at different salinity levels. *Plant Soil* 273, 245–256.
5. Asghari, H.R. (2008). Vesicular-arbuscular (VA) mycorrhizae improve salinity tolerance in pre-inoculation subterranean clover (*Trifolium subterraneum*) seedlings. *Int. J. Plant Production* 2,243-256.
6. Basak, H., Demdr, K., Kasim, R., and Okay, F.Y. (2011). The effect of endo-mycorrhiza (VAM) treatment on growth of tomato seedling grown under saline conditions. *Afr.* *J Agri. Res.* 6, 2532-2538.
7. Benothmane, A. (2011). Mycorrhizal colonization and growth characteristics of salt stressed *Solanum lycopersicum* L. Department of Biology, Faculty of Science, University of Ottawa.
8. Bharti, N., Baghel, S., Barnawal, D., Yadav, A., and Kalra, A. (2013). The greater effectiveness of *Glomus mosseae* and *Glomus intraradices* in improving productivity, oil content and tolerance of salt stressed menthol mint *(Mentha arvensis).* [*J. Sci. Food Agri*](http://onlinelibrary.wiley.com/journal/10.1002/%28ISSN%291097-0010). 93, 2154–2161.
9. Borde, M., Dudhane, M., and Jite, P.K. (2010). AM fungi influences the photosynthetic activity, growth and antioxidant enzymes in *Allium sativum* L. under salinity condition. *Not. Sci. Biol*. 2, 64-71.
10. Borde, M., Dudhane, M., and Jite, P.K. (2011). Growth photosynthetic activity and antioxidant responses of mycorrhizal and non-mycorrhizal bajra (*Pennisetum glaucum*) crop under salinity stress condition. *Crop Protection*, 30, 265−271.
11. Cantrell, I.C., and Linderman, R.G. (2001). Preinoculation of lettuce and onion with VA mycorrhizal fungi reduces deleterious effects of soil salinity. *Plant Soil* 233, 269–281.
12. Dudhane, M., Borde, M., and Jite, P.K. (2011). Effect of Arbuscular Mycorrhizal fungi on growth and antioxidant activity in G*melina arborea* Roxb. under salt stress condition. *Not Sci Biol.*, 3(4), 71-78.
13. Echeverria, M., Scambato, A.A., Sannazzaro, A.I., Maiale, S., Ruiz, O.A., and Menéndez, A.B. (2008). Phenotypic plasticity with respect to salt stress response by *Lotus glaber*: the role of its AM fungal and rhizobial symbionts. *Mycorrhiza*, 18, 317−329.
14. Echeverria, M., Sannazzaro, A.I., Ruiz, O.A., and Menéndez, A.B. (2013). Modulatory effects of *Mesorhizobium tianshanense* and *Glomus intraradices* on plant proline and polyamine levels during early plant response of *Lotus tenuis* to salinity. *Plant Soil*, 364, 69−79.
15. Estrada, B., Aroca, R., Barea, J.M., and Ruiz-Lozano, J.M. (2013a). Native arbuscular mycorrhizal fungi isolated from a saline habitat improved maize antioxidant systems and plant tolerance to salinity. *Plant Science*, 201−202, 42−51.
16. Estrada, B., Barea, J.M., Aroca, R., and Ruiz-Lozano, J.M. (2013b). A native *Glomus intraradices* strain from a Mediterranean saline area exhibits salt tolerance and enhanced symbiotic efficiency with maize plants under salt stress conditions. *Plant Soil*, 366, 333−349.
17. Estrada, B., Aroca, R., Maathuis, J.M, Barea, J.M., and Ruiz-lozano, J.M. (2013c). Arbuscular mycorrhizal fungi native from a Mediterranean saline area enhance maize tolerance to salinity through improved ion homeostasis. *Plant, Cell and Environment,* doi: 10.1111/pce.12082, 1-12.
18. Evelin, H., Giri, B., and Kapoor, R. (2012). Contribution of *Glomus intraradices* inoculation to nutrient acquisition and mitigation of ionic imbalance in NaCl-stressed *Trigonella foenum-graecum*. *Mycorrhiza*, 22, 203–217.
19. Evelin, H., Giri, B., and Kapoor, R. (2013). Ultrastructural evidence for AMF mediated salt stress mitigation in *Trigonella foenum-graecum. Mycorrhiza* 23,71–86.
20. Garg, N., and Baher, N. (2013). Role of arbuscular mycorrhizal symbiosis in proline biosynthesis and metabolism of *Cicer arietinum* L. (Chickpea) genotypes under salt stress. *J Plant Growth Regul*, DOI 10.1007/s00344-013-9346-4, 22 June 2013.
21. Garg, N., and Chandel, S. (2011). The effects of salinity on nitrogen fixation and trehalose Metabolism in mycorrhizal C*ajanus cajan* (L.) Mill sp. Plants. *J Plant Growth Regul*., 30, 490–503.
22. Garg, N., and Chandel, S. (2012). Role of Arbuscular Mycorrhizal (AM) fungi on growth, Cadmium uptake, osmolyte, and phytochelatin synthesis in *Cajanus cajan* (L.) Millsp. under NaCl and Cd stresses. *J. Plant Growth Regul*. 31, 292–308.
23. Giri, B., Kapoor, R., and Mukerji, K. G. (2003). Influence of arbuscular mycorrhizal fungi and salinity on growth, biomass, and mineral nutrition of *Acacia auriculiformis*. *Biol. Fertil. Soils* 38, 170–175.
24. Giri, B., and Mukerji, K.G. (2004). Mycorrhizal inoculant alleviates salt stress in *Sesbania aegyptiaca* and *Sesbania grandiflora* under field conditions: evidence for reduced sodium and improved magnesium uptake. *Mycorrhiza*, 14, 307–312.
25. Giri, B., Kapoor, R., and Mukerji, K.G. (2007). Improved Tolerance of Acacia nilotica to Salt Stress by Arbuscular Mycorrhiza, Glomus fasciculatum may be Partly Related to Elevated K/Na Ratios in Root and Shoot Tissues. *Microb. Ecol.* 54, 753–760.
26. Gupta, N., and Rautaray, S. (2005). Growth and development of AM fungi and maize under salt and acid stress. *Acta Agriculturae Scandinavica Section B-Soil and Plant Science*, 55, 151-157.
27. Hajiboland, R., Aliasgharzadeh, N., Laiegh, S.F., and Poschenrieder, C. (2010). Colonization with arbuscular mycorrhizal fungi improves salinity tolerance of tomato (*Solanum lycopersicum* L.) plants. *Plant Soil*, 331, 313–327.
28. Hatimi, A. (1999). Effect of salinity on the association between root symbionts and *Acacia cyanophylla* Lind.: growth and nutrition. *Plant Soil* 216, 93–101.
29. He, Z., and Huang, Z. (2013). Expression analysis of lenhx1 gene in mycorrhizal tomato under salt stress. *J. Microbiol.* 51, 100–104.
30. Huang, J.C., Lai, W.A., Singh, S., Hameed, A., and Young, C.C. (2013). Response of mycorrhizal hybrid tomato cultivars under saline stress. *J. Soil Sci. Plant Nutri.* 13, 469-484.
31. Jahromi, F., Aroca, R., Porcel, R., and Ruiz-Lozano, J.M. (2008). Influence of salinity on the in vitro development of *Glomus intraradices* and on the *in vivo* physiological and molecular responses of mycorrhizal lettuce plants. *Microb. Ecol.* 55, 45–53.
32. Jalaluddin, M. (1999). Effect of VAM fungus (*Glomus intraradices*) on the growth of Soghum, Maize, Cotton and *Pennisetum* under salt stress. *Pak. J. Bot.* 25, 215-218.
33. Kadian, N., Yadav, K., Badda, N., and Aggarwal, A. (2013a). Application of Arbuscular Mycorrrhizal Fungi in improving Growth and Nutrient of *Cyamopsis etragonoloba* (L.) Taub. under Saline Soil. *Int. J. Agron. Plant Production*. 4, 2796-2805.
34. Kadian, N., Yadav, K., Badda, N., and Aggarwal, A. (2013b). AM Fungi Ameliorates Growth, Yield and Nutrient Uptake in *Cicer arietinum* L. Under Salt Stress. *Russ. Agri. Sci.* 39, 321–329.
35. Kashyap, S., and Sharma, S. (2005). Role of bioinoculants and auxin in development of salt tolerant *Mentha arvensis*. *Hort. Sci*. 32, 31–41.
36. Kashyap, S., and Sharma, S. (2006). In vitro selection of salt tolerant *Morus alba* and its field performance with bioinoculants. *Hort. Sci*., 33(2): 77–86.
37. Kohler, J., Caravaca, F., and Roldán, A. (2010). An AM fungus and a PGPR intensify the adverse effects of salinity on the stability of rhizosphere soil aggregates of *Lactuca sativa.* *Soil Biol. Biochem.* 42, 429−434.
38. Kumar, A., Sharma, S., and Mishra, S. (2010). Influence of Arbuscular Mycorrhizal (AM) Fungi and Salinity on Seedling Growth, Solute Accumulation, and Mycorrhizal Dependency of *Jatropha curcas* L. *J. Plant Growth Regul*. 29, 297–306.
39. Li, T., Liu, R.J., He, X.H., and Wang, B.S. (2012). Enhancement of superoxide dismutase and catalase activities and salt tolerance of euhalophyte S*uaeda salsa L*. by mycorrhizal fungus G*lomus mosseae. Pedosphere,* 22, 217–224.
40. McHugh JM., and Dighton, J. (2004). Influence of Mycorrhizal Inoculation, Inundation Period, Salinity, and Phosphorus Availability on the Growth of Two Salt Marsh Grasses, *Spartina alterniflora* Lois. and *Spartina cynosuroides* (L.) Roth., in Nursery Systems. *Restoration Ecol.* 12, 533–545.
41. Muok, B.O., and Ishii, T. (2006). Effect of Arbuscular Mycorrhizal fungi on tree growth and nutrient uptake of *Sclerocarya birrea* under water stress, salt stress and flooding. *J. Japan. Soc. Hort. Sci.*75, 26–31.
42. Navarroa, A., Elia, A., Conversa, G., Campia, P., and Mastrorilli, M. (2012). Potted mycorrhizal carnation plants and saline stress: Growth, quality and nutritional plant responses. *Scientia Horticulturae* 140, 131–139.
43. Navarroa, J.M., Pérez-Torneroa, O., and Morte, A. (2013). Alleviation of salt stress in citrus seedlings inoculated with arbuscular mycorrhizal fungi depends on the rootstock salt tolerance. *Journal of Plant Physiology*, <http://dx.doi.org/10.1016/j.jplph.2013.06.006>
44. Peng, J., Li, Y., Shi, P., Chen, X., Lin, H., and Zhao, B. (2011). The differential behavior of arbuscular mycorrhizal fungi in interaction with *Astragalus sinicus* L. under salt stress. *Mycorrhiza*, 21, 27–33.
45. Patel, D., and Saraf, M. (2013). Influence of soil ameliorants and microflora on induction of antioxidant enzymes and growth promotion of *Jatropha curcas* L. under saline condition. *Euro. J. Soil Biol.* 55, 47-54.
46. Qun et al., (2010). Arbuscular mycorrhizal alleviated ion toxicity, oxidative damage, and enhance osmotic adjustment in tomato subjected to NaCl stress. *J. Agric. Environ. Sci*., 7(6), 676-683.
47. Sannazzaro, A.I., Ruiz, O.A., Alberto, E.O., and Mene´ndez, A.B. (2006). Alleviation of salt stress in *Lotus glaber* by *Glomus intraradices*. *Plant Soil*, 285, 279–287.
48. Sannazzaro, A.I., Echeverrı´a, M., Alberto´, E.O., Ruiz, O.A. & Mene´ndez, A.B. (2007). Modulation of polyamine balance in *Lotus glaber* by salinity and arbuscular mycorrhiza. *Plant Physiology and Biochemistry*, 45, 39-46.
49. Scambato, A.A., Echeverria, M., Sansberro, P., Ruiz1, O.S., and Menéndez, A.B. (2011). *Glomus intraradices* improved salt tolerance in *Prosopis alba* seedlings by improving water use efficiency and shoot water content. *Braz. J. Plant Physiol*. 22, 285-289.
50. Selvakumar, G., and Thamizhiniyan, P. (2011). The effect of the arbuscular mycorrhizal (AM) fungus G*lomus intraradices* on the growth and yield of Chilli (C*apsicum annuum L*.) under salinity stress. *World Appl. Sci. J.* 14 , 1209-1214.
51. Sheng, M., Tang, M., Chen, H., Yang, B., Zhang, F., and Huang, Y. (2008). Influence of arbuscular mycorrhizae on photosynthesis and water status of maize plants under salt stress. *Mycorrhiza*, 18, 287–296.
52. Sheng, M., Tang, M., Zhang, F., and Huang, Y. (2011). Influence of arbuscular mycorrhiza on organic solutes in maize leaves under salt stress. *Mycorrhiza* 21, 423–430.
53. Talaat, N.B., and Shawky, B.T. (2013). Modulation of nutrient acquisition and polyamine pool in salt-stressed wheat (*Triticum aestivum* L.) plants inoculated with arbuscular mycorrhizal fungi. *Acta Physiol Plant*, 35, 2601–2610.
54. Talaat, N.B., and Shawky, B.T. (2014). Protective effects of arbuscular mycorrhizal fungi on wheat(*Triticum aestivum* L.) plants exposed to salinity. *Environ. Exp. Bot.* 98, 20– 31.
55. Tian, C.Y., Feng, G., Li, X.L., and Zhang, F.S. (2004). Different effects of arbuscular mycorrhizal fungal isolates from saline or non-saline soil on salinity tolerance of plants. *Appl. Soil Ecol.* 26, 143–148.
56. Tsang, A., and Maun. M.A. (1999). Mycorrhizal fungi increase salt tolerance of *Strophostyles helvola* in coastal foredunes. *Plant Ecology* 144, 159–166.
57. Wu, Q.S., Zou, Y.N., and Heb, X.H. (2013). Mycorrhizal symbiosis enhances tolerance to NaCl stress through selective absorption but not selective transport of K+ over Na+ in trifoliate orange. *Scientia Horticulturae* 160, 366–374.
58. Zhang Y.F., Wang P., Yang, Y.F., Bi, Q., Tian, S.Y., and Shi. X.W. (2011). Arbuscular mycorrhizal fungi improve reestablishment of *Leymus chinensis* in bare saline-alkaline soil: Implication on vegetation restoration of extremely degraded land. *J. Arid Environ.* 75, 773-778.
59. Zhi, H., Chao-xing, H., Zhong-qun, H., Zhi-rong, Z., and Zhi-bin, Z. (2010). The Effects of Arbuscular Mycorrhizal Fungi on Reactive Oxyradical Scavenging System of Tomato Under Salt Tolerance. *Agri. Sci. China*, 9, 1150-1159.
60. Zou, Y.N., and Wu, Q.S. (2011). Efficiencies of five arbuscular mycorrhizal fungi in alleviating salt stress of trifoliate orange. *Int. J. Agric. Biol*. 13, 991–995.
