# Supplementary material for: Mycorrhizal Symbiotic Efficiency on C3 and C4 Plants under Salinity Stress – A Meta-Analysis
Source: Front Microbiol. 2016 Aug 11;7:1246. doi: 10.3389/fmicb.2016.01246 (PMC4981042; doi:10.3389/fmicb.2016.01246)
Supplement: Supplementary file 2 [file Data_Sheet_2.DOC]

**Supporting Information**

**Mycorrhizal symbiotic efficiency on C_3_ and C_4_ plants under salinity stress: A meta-analysis**

Murugesan Chandrasekaran, Kiyoon Kim, Ramasamy Krishnamoorthy, Denver Walitan, Subbiah Sundaram, Manoharan Melvin Joe, Gopal Selvakumar, Suijin Hu, Sang-Hyon Oh, Tongmin Sa

**SI Results**

**1. Study Coding Glossary**

2. **Publication bias**

1. C_3_ and C_4_ dataset

2. Response variable datasets

3. **Details of Sensitivity analysis**

4. **Random-effects categorical model analysis**

1. Overall categorical analysis under salt stress

2. Overall categorical analysis under normal condition

**5. Correlation analysis**

Relationship between K uptake and soil salinity

**6. Supplementary Tables**

1. Overall **s**ignificance of factors analyzed in the categorical analyses based on the significance of the variation among categories (Q_B_) and the amount of the total variation (Q_T_) described by Q_B_/Q_T_ under salt stress

2. **(**a) Summary of overall heterogeneity analysis (b) Summary of the overall publication bias analysis

**1. Study Coding Glossary**

NUMBER OF STUDIES: Number of studies included in the meta-analysis

STUDY CODE: Assigned numerical ID for publication included

PUBLICATION: First author and year of publication, e.g. Evelin *et al*., 2012

AMF SPECICES: Generic and species name of treatment organism, e.g. *Glomus intraradices*

PLANT SPECIES: Generic and species name of plant studied, e.g. *Lycopersicon esculentum*

PLANT FAMILY: Taxonomy of different plant species based on broad plant family

PHOTOSYNTHETIC TYPES: C3 and C4 plants response to AMF inoculation

PLANT GROUP: Monocotyledonous and dicotyledonous plants

PLANT DURATION: The effect of growth variables, such as annul and perennial herb, woody and grass

GROWTH HABIT: AMF inoculation responses to plant functional groups such as herb, forb, shrub, and trees

EXPERIMENTAL CONDITION: The effect of symbiosis different among study locations, (field or greenhouse)

SOIL TYPE: The effects of different soil types on AMF inoculation response under salinity stress

DEGREE OF STRESS: The effect of stress level experienced by plant in dS/m

DURATION: Experimental days/weeks/months.

RESPONSE VARIABLE: Measures were grouped into categories that represent shoot, root and total dry weight, N, P, K and Na uptake, and proline accumulation

SAMPLE SIZE: Number of replicates used for the experimental analysis

Xc: Control mean; SDc: Control standard deviation

Xe: Experimental mean; SDe: Experimental standard deviation

lnR: log response ratio

Var(lnR): variance of log response ratio

**2. Publication bias**

We tested our datasets for publication bias by plotting the effect size lnR against the sample size (replicates) and variance (within-study variance; Egger et al., 1997).


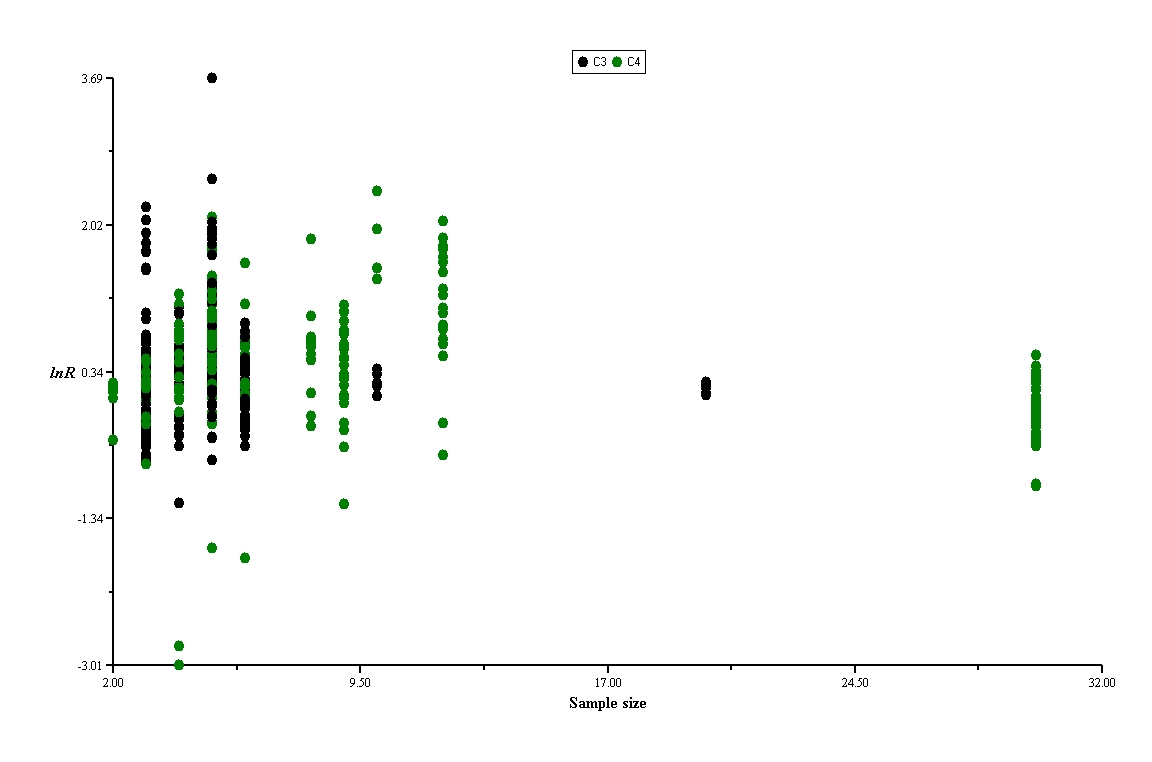

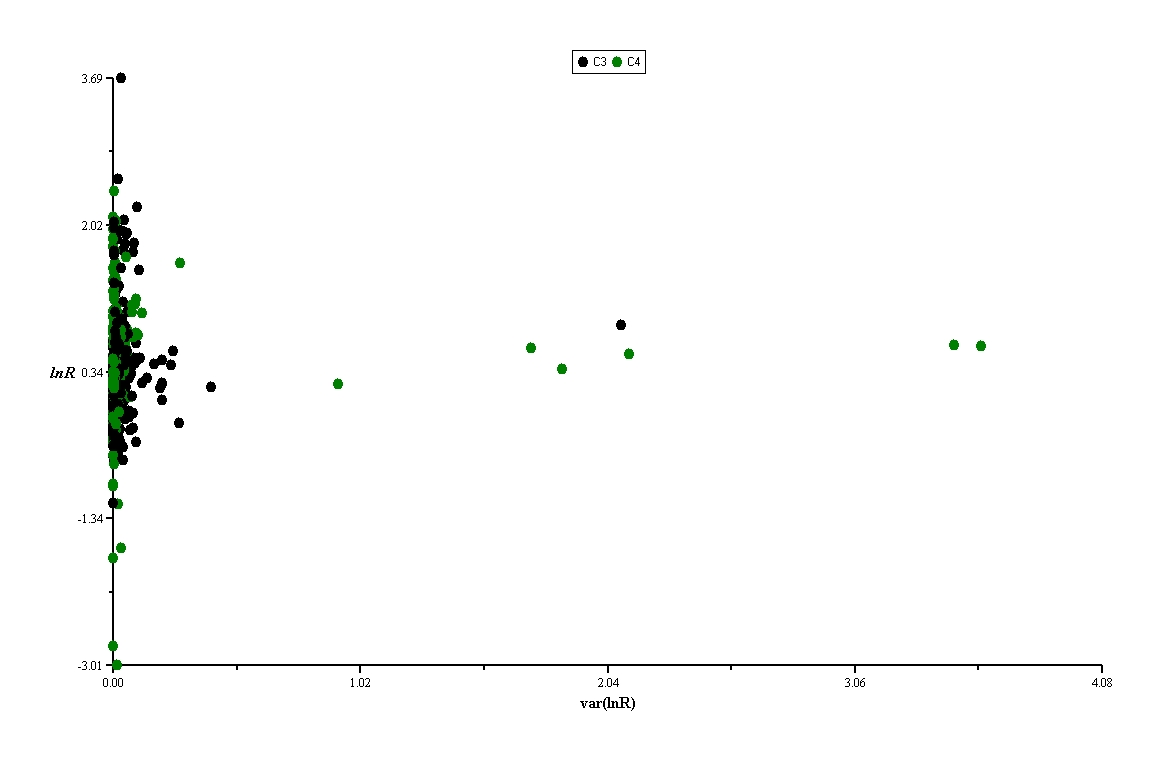


**S1 Fig.** Scatterplots of effect size against sample size (replicates) and sample variance for lnR, respectively, for *C_3_*, and *C_4_* dataset.


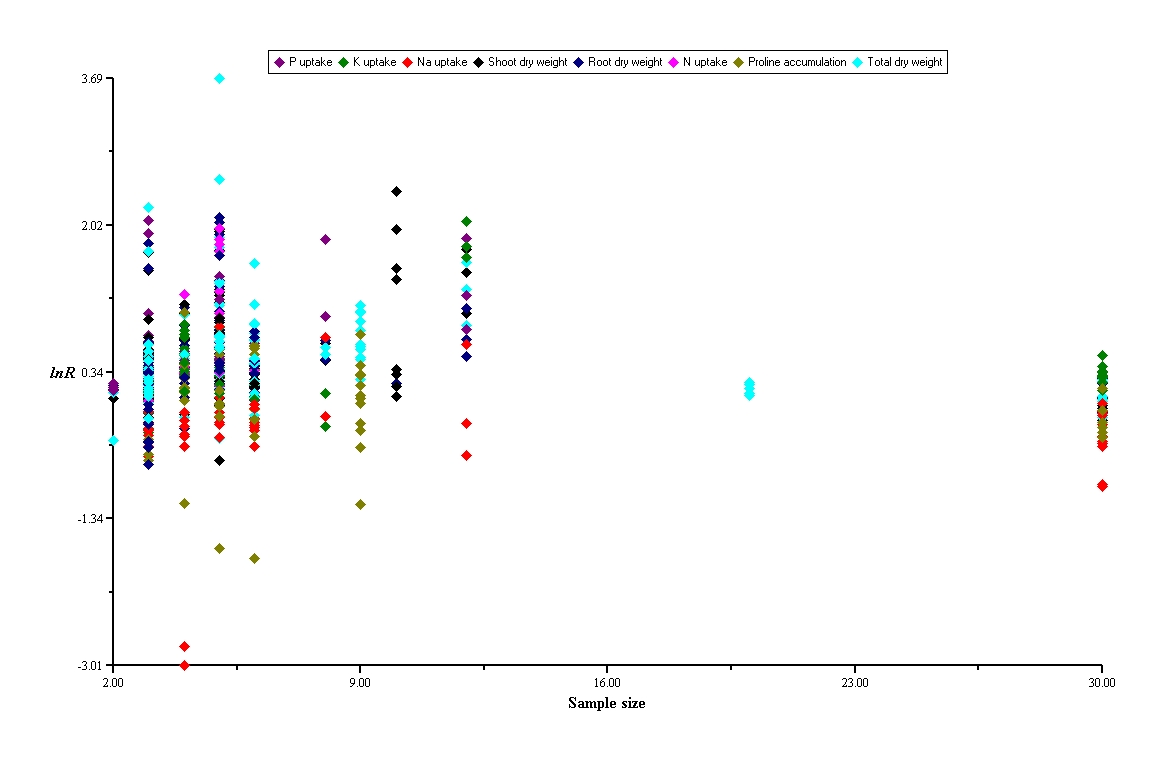

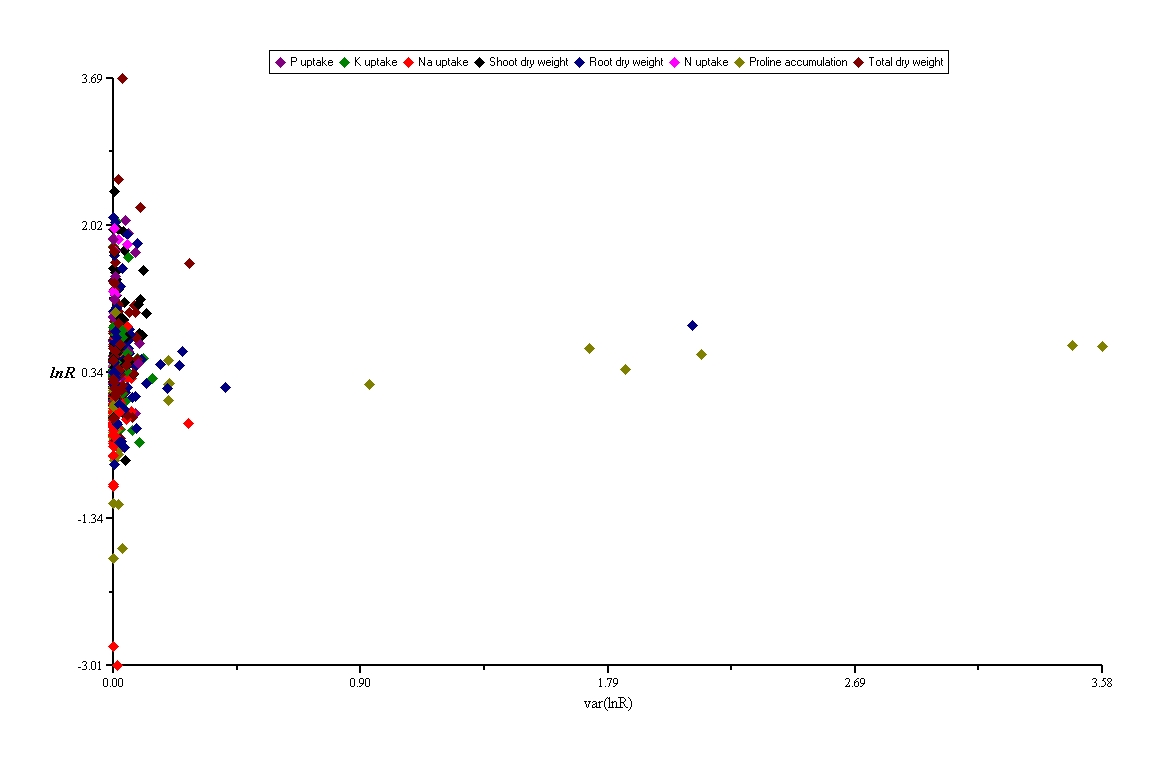
There were patterns suggesting the existence of a publication bias, as would be evident by funnel symmetry based on variance (Nagakawa and Santos, 2012)

**S2 Fig.** Scatterplots of effect size against sample size (replicates) and sample variance for lnR, respectively, for *P uptake*, *K uptake*, *Na uptake,* *shoot dry weight*, *root dry weight*, *N uptake*, *total dry weight* and *Proline accumulation* dataset.

**3. Details of Sensitivity analysis**

A sensitivity analysis was conducted to test for any disproportional impact on studies. However, we only applied this procedure on independent categorical variables significantly affecting AMF inoculation under salt stress. The sensitivity analysis was done in MetaWin by sequentially excluding one study at a time from the dataset. After excluding a study, a new random effects meta-analysis was performed and the effect size estimate and 95% BS CIs were compared with those of the complete dataset. Effect size estimates and 95% BS CIs for each level of the significant categorical independent variables were also investigated.

If the BS CIs did not include the effect size estimate of the complete dataset, then this specific study had a disproportional impact. Consequently, the meta-analysis of the complete dataset had to be repeated without this specific study.


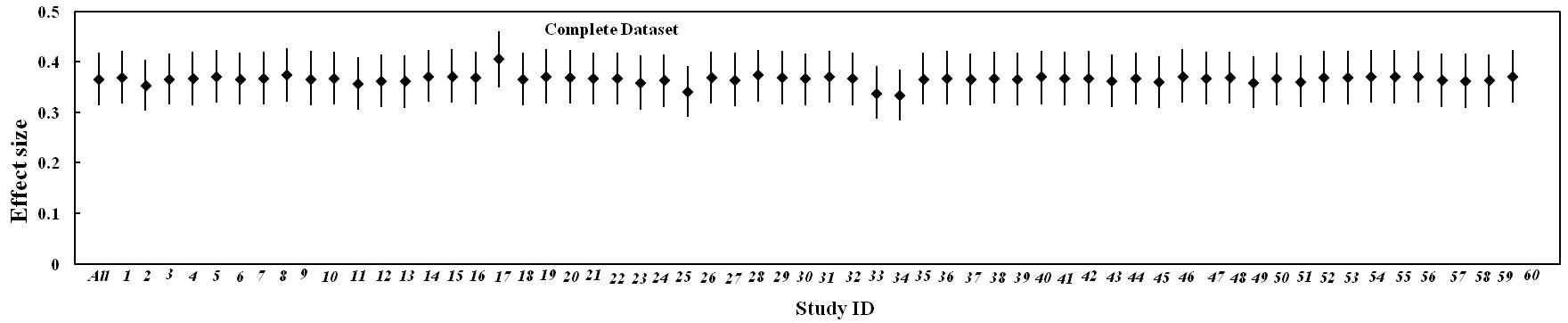


**S3 Fig.** Sensitivity analysis of complete dataset

**S4 Fig.** Sensitivity analysis of C_3_ dataset

**S5 Fig.** Sensitivity analysis of C_4_ dataset

**4. Random-effects categorical model analysis**

**
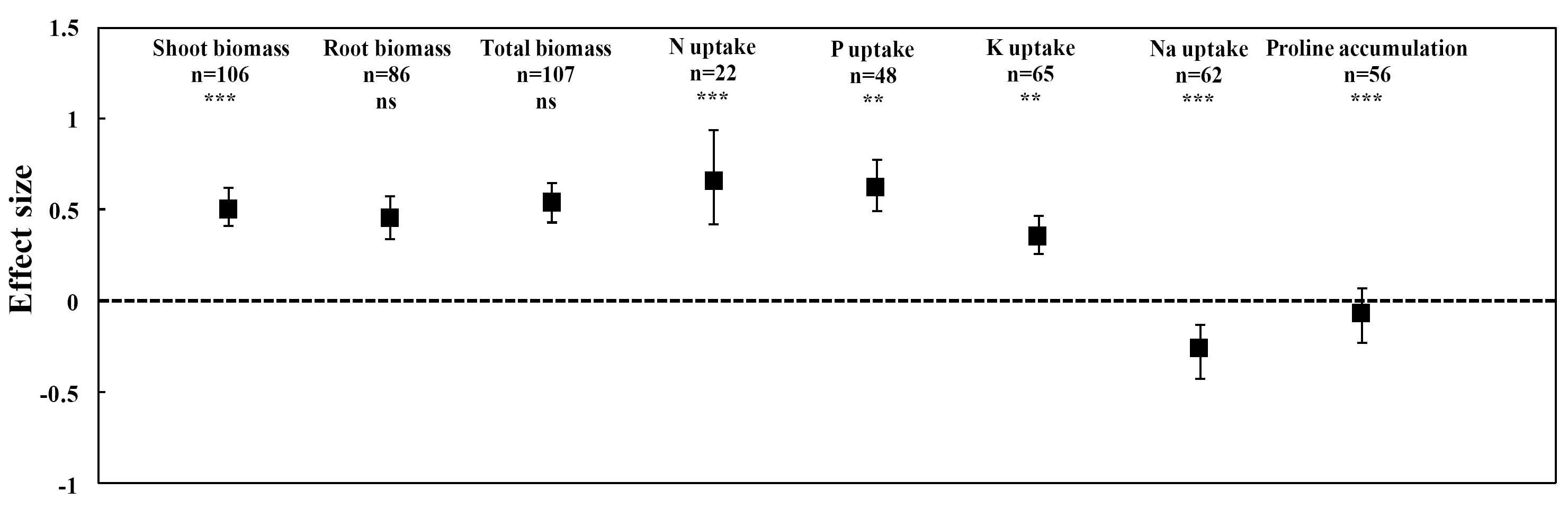
**

**S6 Fig.** **Arbuscular mycorrhizal fungi inoculation responses of plant under salt stress.** Error Bars are means ± bias CIs. Where the bias CIs do not overlap the horizontal dashed lines, the effect size for a parameter is significant at *P*<0.05. All effect sizes is differed significantly from zero (chi-square tests, ***P<0.001, **P<0.01, ns = P>0.05). N=number of studies included in the meta-analysis.

**
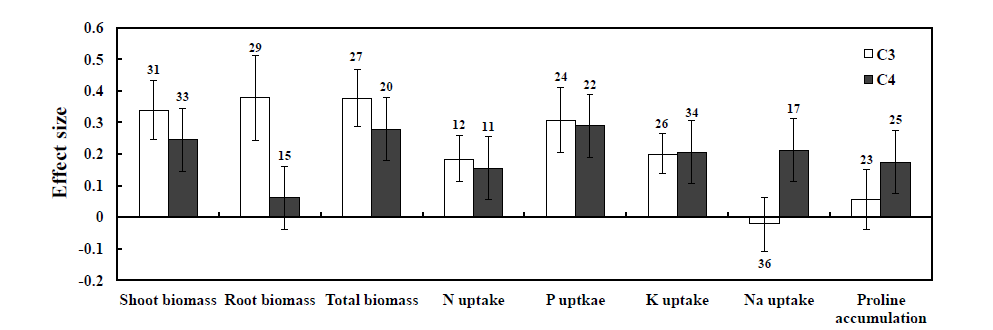
**

**S7 Fig. Comparative photosynthetic growth and nutrient uptake responses of AMF-inoculated plants under normal condition.** Error bars are means±bias CIs. Where the CIs do not overlap the horizontal dashed lines, the effect size for a parameter is significant at *P*<0.05. A number of studies were included for the meta-analysis mentioned above the bar.

**5. Correlation analysis**

We tested the relationship of lnR (K uptake) and its level of salinity. We could detect significant relationship.


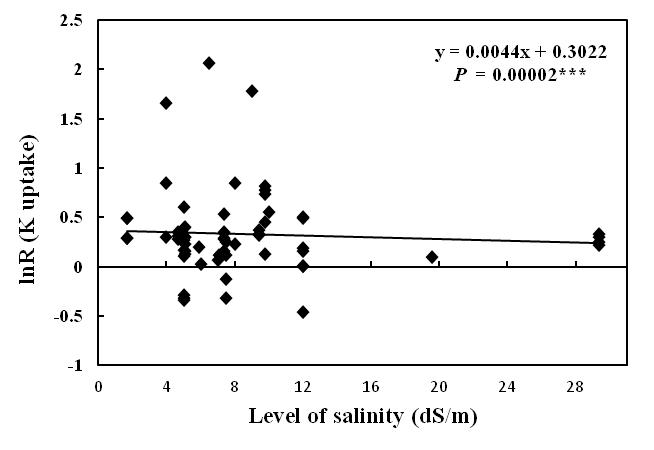


**S8 Fig.** Relationship of the effect size lnR (K uptake) and level salinity for C_3_ and C_4_ dataset. There was significant relationship detectable.

**6. Supplementary Tables**

**S1 Table** Overall **s**ignificance of factors analyzed in the categorical analyses based on the significance of the variation among categories (Q_B_) and the amount of the total variation (Q_T_) described by Q_B_/Q_T_ under salt stress

| **Categorical independent variable** | **Q_B_** | **Q_B_/Q_T_** | ***P* random** |
| --- | --- | --- | --- |
| Response variable | 216.5196 | 0.2127 | 0.0002 |
| AMF species | 85.5185 | 0.0917 | 0.0004 |
| AMF inoculation | 5.9067 | 0.007 | 0.0460 |
| Plant species | 401.8026 | 0.3126 | 0.0002 |
| Photosynthetic types | 2.6797 | 0.0034 | 0.1718 |
| Plant types | 30.9225 | 0.0362 | 0.0002 |
| Plat family | 114.0590 | 0.1124 | 0.0002 |
| Plant duration | 68.1566 | 0.0735 | 0.0002 |
| Plant functional groups | 92.6814 | 0.09676 | 0.0002 |
| Experimental condition | 0.0107 | 1.3746 | 0.9306 |
| Level of salinity | 186.5152 | 0.1844 | 0.0002 |
| Degree of salinity | 10.3659 | 0.0123 | 0.0276 |
| Experimental duration | 14.6553 | 0.01876 | 0.0068 |
| Soil type | 128.3481 | 0.1377 | 0.0124 |

**S2aTable.** Summary of overall heterogeneity analysis

| **Response variable** | **Effect size** | **df** | **Bias CI** | **Q_T_** | **Q_T_(*P*)** |
| --- | --- | --- | --- | --- | --- |
| **All studies** | 0.3643 | 551 | 0.3131 to 0.4171 | 782.0307 | 0.0000 |
| **C_3_ species** | 0.4024 | 223 | 0.3246 to 0.4891 | 604.698 | 0.0000 |
| **C_4_ species** | 0.3354 | 327 | 0.2673 to 0.4026 | 408.467 | 0.0014 |
| **Shoot biomass** | 0.5038 | 105 | 0.4077 to 0.6163 | 262.936 | 0.0000 |
| **Root biomass** | 0.4567 | 85 | 0.3431 to 0.5799 | 40.501 | 0.9999 |
| **Total biomass** | 0.5418 | 106 | 0.4357 to 0.6539 | 123.345 | 0.1484 |
| **N uptake** | 0.6467 | 21 | 0.4131 to 0.9244 | 83.1617 | 0.0000 |
| **P uptake** | 0.6128 | 47 | 0.4948 to 0.7789 | 62.4040 | 0.0456 |
| **K uptake** | 0.3585 | 64 | 0.2661 to 0.4707 | 101.221 | 0.0020 |
| **Na uptake** | -0.2571 | 61 | -0.4300 to -0.1266 | 201.639 | 0.0000 |
| **Proline accumulation** | -0.0703 | 55 | -0.2237 to 0.0685 | 164.799 | 0.0000 |

**S2b Table.** Summary of the overall publication bias analysis

| **Response variable** | **Spearman rho rank** | ***P*** | **Fail safe calculation** | **Rosenthal's Method** |
| --- | --- | --- | --- | --- |
| **All studies** | 0.860 | 0.0002 | 2770 | 55021.6 |
| **C_3_ species** | 0.0140 | 0.8341 |  |  |
| **C_4_ species** | -0.100 | 0.0696 |  |  |
| **Shoot biomass** | -0.050 | 0.6113 |  |  |
| **Root biomass** | -0.005 | 0.9632 |  |  |
| **Total biomass** | -0.063 | 0.0226 | 545 | 382610.4 |
| **N uptake** | 0.711 | 0.0002 | 120 | 36257.2 |
| **P uptake** | 0.301 | 0.0378 | 250 | 85954.8 |
| **K uptake** | -0.168 | 0.1799 |  |  |
| **Na uptake** | 0.081 | 0.5319 |  |  |
| **Proline accumulation** | -0.149 | 0.2723 |  |  |

Significant Spearman’s rho (*P* <0.05) in bold indicates publication bias; fail-safe calculation: 5*n* + 10 (*n* = number of studies); When fail-safe calculation < Fail-safe Rosenthal’s method: publication bias can be safely ignored.
